# Perception and readiness of health professions students about interprofessional education in Kuwait

**DOI:** 10.1186/s12909-026-08622-z

**Published:** 2026-01-19

**Authors:** Anwar B. Almutairi, Yasmeen Mezil, Zainab A. Jasem, Bruce Wainman

**Affiliations:** 1 Physical Therapy Department, Faculty of Allied Health Sciences, Kuwait University, P.O. Box 5969, Jabriya, Safat 13060 Kuwait; 2 Department of Anatomy and Genetics, College of Medicine, Alfaisal University, Riyadh, Kingdom of Saudi Arabia; 3 Occupational Therapy Department, Faculty of Allied Health Sciences, Kuwait University, Kuwait, Safat, Kuwait; 4 Department of Pathology and Molecular Medicine, Faculty of Health Sciences, McMaster University, Hamilton, ON Canada

**Keywords:** Healthcare profession, Readiness, Medical education, Higher education, Professional development, Eastern mediterranean countries

## Abstract

**Background:**

Interprofessional education (IPE) is important for developing interdisciplinary partnerships that improve healthcare delivery. IPE is currently not a common practice in healthcare professions (HP) programs in Middle Eastern countries.

**Objective:**

The aim of this study is to assess the perceptions and attitudes of HP students toward IPE across multiple programs at Kuwait.

**Methods:**

This was an exploratory, cross-sectional, quantitative, comparative study of 1,162 full-time HP program students ≥ 18 years old. The Interdisciplinary Education Perception Scale (IEPS) and Readiness for Interprofessional Learning Scale (RIPLS) were utilized.

**Results:**

IEPS scores were significantly different among different years of study (*p* = 0.027). RIPLS scores were significantly different among different academic HP programs (*p* = 0.042). The IEPS subscales, perception of actual cooperation and perceived need for cooperation, were significantly different among different HP programs (*p* = 0.005). RIPLS subscales, teamwork and collaboration (*p* = 0.024), and roles and responsibilities (*p* = 0.016), were shown to be significantly different among academic years.

**Conclusion:**

Higher IEPS scores among upper-year students emphasize the role of clinical exposure in enhancing perceptions of interdisciplinary collaboration, while RIPLS results suggest that program-specific curricula shape readiness for interprofessional practice. These findings underscore the need for structured IPE experiences tailored to academic stages and professional contexts to prepare collaborative healthcare professionals.

## Introduction

 Optimization of patient care is dependent on the effective communication between healthcare professionals (HP) and teams [12]. Interprofessional collaboration is integral for the development of a comprehensive healthcare team. This communication between healthcare providers should be fostered early on in the careers of healthcare professionals, preferably during their educational programs [2, 14, 18]. Interprofessional education (IPE) is therefore an important building block for developing future interdisciplinary partnerships that would improve healthcare delivery. IPE is a pedagogical method that prepares HP students or professional individuals in different professional programs and work settings to work in a team and seek collaborations to ensure optimal healthcare services [20].

IPE evaluation can be implemented across several levels [6]. Student perception, attitude, and readiness is the foundational level ahead of IPE implementation [9, 14]. This exploration can be conducted using either qualitative or quantitative tools; the Interprofessional Socialization and Valuing Scale is one tool that was developed to examine students and health professionals readiness to function as a team [13]. Another widely used tool for student readiness is the Readiness for Interprofessional Learning Scale (RIPLS) which assesses student readiness toward IPE [5, 20]. Regarding perception toward IPE, the Interdisciplinary Education Perception Scale (IEPS) is a tool that is used to assess the perception of HP students toward IPE. Evaluation during and immediately after IPE can focus on the areas of knowledge, skill, and beliefs [9, 22]. These assessments can be used by the students themselves who self-assess each construct, or conducted by an observer who rates the learner, or a combination of these sources of feedback can be implemented [9].

In Middle Eastern countries, a few studies have assessed HP student perception toward IPE. In two studies conducted in Saudi Arabia, the majority of allied health students demonstrated positive attitudes and exhibited readiness towards IPE [3, 25]. Similarly in a study that encompassed a broader number of countries in the East, pharmacy students in Saudi Arabia, Bangladesh, and Malaysia demonstrated positive trends in their perception and attitudes towards IPE [5]. A mixed methods design was employed in Qatar to assess the perception, readiness, and IPE activity type preference of students toward IPE [11]. The study was conducted with pharmacy students in Qatar who revealed that they had positive perceptions toward IPE and high rates of readiness towards it. Although results appear promising, only students from one healthcare profession were examined in each of these studies. The examination of students from each program at a time is counterintuitive given that these studies are ostensibly about inter-professionalism and an inclusive study design would better represent the views of students from different healthcare professions. In a study conducted at the Health Sciences Center (HSC) with students at Kuwait University (i.e., dentistry, pharmacy, medicine, and allied health programs) positive attitudes toward IPE were observed [15]. This study was conducted using the attitude toward health care teams (ATHCT) scale and the readiness for interprofessional learning scale (RIPLS).

A gap continues to exist as no previous studies have explored attitudes across the entire range of HP students, including underrepresented professions, despite the need for cohesive teamwork to lead to effective collaboration between health-related disciplines. A comprehensive assessment of the perception and readiness of students in all licensing health care professions in Kuwait paves the way for the effective implementation of IPE. The aim of this study is to address the existing gap by conducting a thorough assessment of the perceptions and readiness of all HP students (including nursing, speech therapy, nuclear medicine, nutrition) toward IPE across a range of bachelor’s level programs in Kuwait. The representation from all HP programs mirrors the structure of interprofessional teams in the healthcare system in this region, therefore the inclusion of perceptions and readiness for IPE from all programs is critical for its implementation.

## Methods

### Study design

This is an exploratory, cross-sectional, quantitative, comparative study.

### Participants

Students from different HP programs in the State of Kuwait: medicine (MD), physical therapy (PT), occupational therapy (OT), radiological sciences (RS), medical laboratory sciences (MLS), speech therapy (ST), health informatics (HI), nursing (NR), dentistry (DS), pharmacy (Ph), and nutrition (NI)) were approached. Students across all levels (first year (Y1) students up to seventh year (Y7) students) were invited to participate in this study. Stratified sampling was utilized in this study based on HP program and level in their program. To be included in this study, participants had to be enrolled in a full-time HP program in Kuwait and ≥ 18 years old. All HP programs are part of Kuwait University (KU), except for the NR program which is currently a part of the Public Authority of Applied Education and Training (PAAET) institution. The MD, PT, OT, RS, MLS, HI, DS, and Ph are at the HSC of KU (Al-Jabriya Campus). ST and NI are programs offered by the College of Life Sciences at KU (Al-Shedadiya campus). MD, DS, and Ph, which are seven-years programs (with the first 4 years being a Bachelor of Basic Medical Science and the remaining three years are specialization years in the respective profession), while the remaining programs are only four years in duration.

### Procedure

The study was approved by the Kuwait University HSC ethics committee (VDR/EC – 495), in accordance with the Declaration of Helsinki and was conducted between November 2023 and July 2024. Study announcements were displayed on advertisement screens throughout the various campuses, and a mass email was sent to students in HP programs inviting them to participate. Written consent form was obtained from the participants. Afterwards, participants completed an online survey that included three parts: (1) demographic questions (i.e., age, gender, year of study and major of study), (2) the IEPS questionnaire [5, 20], and (3) the RIPLS questionnaire [3, 20]. Clinical trial number is not applicable.

The IEPS and RIPLS questionnaires are validated tools designed to assess students’ perception and readiness to be involved in interprofessional/interdisciplinary education. IEPS consists of 12 questions using a Likert scale ranging between 1 (strongly disagree) and 6 (strongly agree). Its subscales are: (1) perception of actual cooperation (derived from items 1–5), (2) perceived need for cooperation (derived from items 6–7), and (3) competency and autonomy (derived from items 8–12) [12]. The RIPLS consists of 18 questions using a Likert scale ranging between 1 (strongly disagree) and 5 (strongly agree). Its subscales are: (1) teamwork and collaboration (derived from items 1–9), (2) negative professional identity (derived from items 10–12), (3) positive professional identity (derived from items 13–15), and (4) roles and responsibilities (derived from items 16–18) [12]. Higher scores in IEPS indicate a positive perception of the individuals’ profession and the professions of others while a high score on RIPLS indicates increased readiness towards participating in IPE. Cultural adaptations for students in Kuwait were not made to any of the questionnaires. Finally, students were asked whether or not they had been exposed to an IPE experience, where IPE was defined as a structured opportunity to interact with other healthcare professional students to acquire knowledge, skills, and professional attitudes [7, 12]. Students were offered the choice to complete either one or both questionnaires.

### Data analysis

Data analyses were conducted using IBM^®^ SPSS Statistics for Windows, Version 27.0 (IBM Corp., Armonk, NY, USA). The statistical significance level was accepted as α = 0.05. The Shapiro–Wilk and Kolmogorov–Smirnov tests were used to assess normality of all variables. Descriptive variables were expressed as mean and standard deviations (mean ± SD) for continuous variables and as a count (n) and percentage (%) for categorical variables. Independent t-test and one-way ANOVA tests were used to assess the difference of IEPS and RIPLS means based on demographic variables data. Additionally, one-way ANOVAs followed by post hoc, multiple pairwise comparisons were used to evaluate the differences in the IEPS and RIPLS scores (total and subscale scores) across the health professions programs and years. Moreover, a point-biserial correlation was measured between experience with IPE and the total score followed by correlations between IPE and each subscale score in both tools.

## Results

One thousand one hundred sixty-two students participated in this study. More than half of the participants completed the IEPS, (54.7%, *n* = 636) while 48.11% of the participants completed the RIPLS (*n* = 559). The mean age of the participants was 19.61 ± 5.54. Physical therapy students were the most numerous HP group and comprised 27.45% of the sample (*n* = 319). The second largest participant group were occupational therapy students (11.36%, *n* = 132). More than one-fourth of the sample size were in the second year of their program (26.68%, *n* = 310). The majority of participants were females (64.97%, *n* = 755). There was no significant difference between genders in total scores of both IEPS and RIPLS questionnaires (*p* = 0.781 and *p* = 0.859, respectively) (Table 1).

**Table 1:** Characteristics of the study sample

		Revised Interdisciplinary Education Perception Scale (IEPS)			Revised Readiness for Interprofessional Learning Scale (RIPLS)		
	Total Sample % (*n*)	% (*n*)	Total score	*P*-value*	% (*n*)	Total score	*P*-value*
Overall	100.0(1162)	54.7(636)	52.40(± 11.93)	-	48.11(559)	65.88(± 9.34)	-
Gender							
Male	6.88(80)	9.43(60)	53.15(± 9.94)	0.781	9.66(54)	66.22(± 8.26)	0.859
Female	64.97(755)	83.18(529)	52.71(± 11.87)		83.18(465)	65.98(± 9.27)	
Missing values	28.1(327)	47			40		
Age, (Mean (± SD)	19.61(± 5.54)	72.01(458)	19.6(± 5.54)	0.687	71.91(402)	19.6(± 5.54)	0.346
Academic Health Professional Major							
Physical Therapy	27.45(319)	38.68(246)	50.93(± 11.92)	0.110^**^	40.61(227)	64.79(± 9.18)	**0.042** ^**^
Occupational Therapy	11.36(132)	16.98(108)	52.91(± 12.55)		16.64(93)	64.87(± 12.01)	
Speech/Language Therapy	2.58(30)	2.99(19)	57.15(± 11.80)		2.86(16)	70.69(± 7.14)	
Medicine	8.43(98)	8.65(55)	53.48(± 9.41)		8.23(46)	67.33(± 6.49)	
Dentistry	2.07(24)	2.20(14)	60.35(± 6.08)		2.15(12)	68.75(± 4.71)	
Pharmacy	3.70(43)	4.25(27)	53.92(± 12.23)		3.94(22)	67.00(± 11.30)	
Nursing	1.80(21)	2.51(16)	48.93(± 15.14)		2.68(15)	67.13(± 5.91)	
Radiological Sciences	3.53(41)	3.77(24)	54.41(± 7.512)		3.22(18)	66.17(± 8.82)	
Medical Laboratory Sciences	10.76(125)	13.68(87)	52.61(± 13.48)		13.24(74)	67.20(± 8.46)	
Health Informatics	2.32(27)	2.99(19)	51.37(± 11.90)		3.04(17)	68.76(± 9.67)	
Nuclear Medicine	0.34(4)	0.47(3)	47.67(± 12.66)		0.54(3)	53.00(± 3.61)	
Nutrition	2.75(32)	2.83(18)	53.72(± 9.16)		2.86(16)	66.25(± 5.18)	
Missing values	22.89(266)	-			0	-	
Academic Years							
1 st year	9.12(106)	8.33(53)	53.13(± 11.92)	**0.027** ^**^	7.87(44)	66.64(± 5.75)	0.060^**^
2nd year	26.68(310)	34.43(219)	54.22(± 11.15)		38.81(189)	67.24(± 8.36)	
3rd year	21.86(254)	32.86(209)	51.09(± 12.12)		33.63(188)	64.66(± 9.96)	
4th year	2.67(31)	3.3(21)	54.24(± 11.79)		3.22(18)	68.72(± 5.22)	
5th year	14.29(166)	18.87(120)	50.27(± 12.91)		19.14(107)	64.56(± 11.42)	
6th year	1.72(20)	1.57(10)	57.70(± 5.25)		1.78(10)	68.30(± 4.30)	
7th year	0.51(6)	0.63(4)	51.00(± 12.25)		0.54(3)	67.00(± 3.61)	
Missing values	23.1(269)	-			0	-	
Previous experience with IPE							
Yes	23.84(277)	28.62(182)	50.81(± 13.65)	**0.050**	29.69(166)	64.48(± 11.96)	**0.022**
No	53.10(617)	70.75(450)	53.04(± 11.11)		70.30(393)	66.47(± 7.92)	
Missing values	23.1(268)	2			0		

*P*-value < 0.05 was considered statistically significant

Bold indicates statistically significant comparison

* Calculated using the parametric Independent Samples T Test and ** One Way ANOVA Test

Over half of the participants reported no IPE experience (53.10%, *n* = 617) though 23.1% of the participants did not disclose this information (*n* = 268). IEPS and RIPLS total scores were significantly different among participants with IPE experience (*p* = 0.050 and *p* = 0.022, respectively); Interestingly, with students that reported not having any previous IPE experience scoring higher than the ones that had a previous experience with IPE (Table 1).

The findings of both questionnaires are presented based on program of study and on years of study (i.e. student stage in the program) (Tables 2, 3, 4 and 5). In terms of total score, IEPS scores were significantly different across years of study (*p* = 0.027), with sixth year students scoring the highest. On the other hand, RIPLS scores were significantly different across the academic HP programs (*p* = 0.042), with ST students scoring the highest.

**Table 2:** Descriptive statistics of revised interdisciplinary education perception scale (IEPS) across academic health professional major

	Academic Health Professional Major												
	Physical Therapy	Occupational Therapy	Speech Therapy	Medicine	Dentistry	Pharmacy	Nursing	Radiological Sciences	Medical Laboratory Sciences	Health Informatics	Nuclear Medicine	Nutrition	*P*-value
Overall, % (n)	27.45(319)	11.36(132)	2.58(30)	8.43(98)	2.07(24)	3.70(43)	1.80(21)	3.53(41)	10.76(125)	2.32(27)	0.34(4)	2.75(32)	-
Student distribution %(n)	38.68(246)	16.98(108)	2.99(19)	8.65(55)	2.20(14)	4.25(27)	2.51(16)	3.77(24)	13.68(87)	2.99(19)	0.47(3)	2.83(18)	-
Perception of actual cooperation subscale													
Mean	20.50	21.46	23.63	22.12	27.00	21.59	19.75	21.75	22.08	20.63	18.33	21.39	0.005
SD	5.66	5.96	4.45	5.040	2.83	5.30	7.160	4.475	6.12	6.25	8.39	6.39	
Perceived need for cooperation subscale													
Mean	8.51	8.06	9.32	10.25	7.86	9.26	7.87	8.71	8.02	7.89	7.33	8.67	**0.001**
SD	2.37	2.30	2.31	1.73	2.28	2.63	2.47	1.97	2.45	2.77	0.58	2.00	
Competency and autonomy subscale													
Mean	21.89	23.31	24.21	20.94	25.50	23.07	20.93	23.95	22.51	22.84	22.00	23.66	0.074
SD	5.63	5.96	6.59	5.12	3.48	5.70	6.69	3.581	6.296	5.84	4.00	4.59	
Total IEPS													
Mean	50.93	52.91	57.15	53.48	60.35	53.92	48.93	54.41	52.61	51.37	47.67	53.72	0.110
SD	11.92	2.55	11.80	9.41	6.08	12.23	15.14	7.51	13.48	11.90	12.66	9.16	

*P*-value < 0.05 was considered statistically significant

Bold indicates statistically significant comparison

*SD* Standard deviation

**Table 3:** Descriptive statistics of Revised Interdisciplinary Education Perception Scale (IEPS) across academic year

	Academic Year							
	First year	Second year	Third year	Fourth year	Fifth year	Sixth year	Seventh year	*P*-value
Overall, % (n)	9.12 (106)	26.68 (310)	21.86 (254)	2.67 (31)	14.29 (166)	1.72 (20)	0.51 (6)	-
Student distribution %(n)	9.12 (106)	26.68 (310)	21.86 (254)	2.67 (31)	14.29 (166)	1.72 (20)	0.51 (6)	-
Perception of actual cooperation subscale								
Mean	21.08	22.37	20.69	22.29	20.36	24.10	21.75	**0.013**
SD	5.76	5.37	5.673	6.634	6.35	3.035	6.02	
Perceived need for cooperation subscale								
Mean	8.53	8.36	8.43	10.10	8.63	9.80	9.50	**0.025**
SD	2.57	2.25	2.41	1.58	2.55	2.04	1.91	
Competency and autonomy subscale								
Mean	23.53	23.49	21.86	21.86	21.26	23.80	19.75	**0.006**
SD	5.42	5.38	5.85	5.738	5.98	3.77	7.08	
Total IEPS								
Mean	53.13	54.22	51.09	54.24	50.27	57.70	51.00	**0.027**
SD	11.92	11.15	12.12	11.79	12.91	5.25	12.25	

*P*-value < 0.05 was considered statistically significant

Bold indicates statistically significant comparison

*SD* Standard deviation

For the IEPS subscales, both perception of actual cooperation and perceived need for cooperation subscales were significantly different among the HP programs (*p* = 0.005). Post hoc comparisons demonstrated that dentistry students scored significantly higher than students in other programs in the perception of actual cooperation subscale (*p* = 0.032), while medical students scored the highest in the perceived need for cooperation subscale (*p* = 0.029) (Table 2). Across academic years, all subscales of the IEPS were significantly different (perception of actual cooperation (*p* = 0.013); perceived need for cooperation (*p* = 0.025); competency and autonomy, *p* = 0.006). Second-year students scored significantly higher than students in other years on both perception of actual cooperation (*p* = 0.041), and competency and autonomy (*p* = 0.048). Conversely, fourth-year students had the highest score in perceived need for cooperation (*p* = 0.035) (Table 3).

**Table 4:** Descriptive statistics of Revised Readiness for Interprofessional Learning Scale (RIPLS) across academic health professional major

	Academic Health Professional Major													
	Physical Therapy	Occupational Therapy	Speech Therapy	Medicine	Dentistry	Pharmacy	Nursing	Radiological Sciences	Medical Laboratory Sciences	Health Informatics	Nuclear Medicine	Nutrition	*P*-value	
Overall, % (n)	27.45(319)	11.36(132)	2.58(30)	8.43(98)	2.07(24)	3.70(43)	1.80(21)	3.53(41)	10.76(125)	2.32(27)	0.34(4)	2.75(32)	-	
Student distribution %(n)	40.61(227)	16.64(93)	2.86(16)	8.23(46)	2.15(12)	3.94(22)	2.68(15)	3.22(18)	13.24(74)	3.04(17)	0.54(3)	2.86(16)	-	
Teamwork and collaboration subscale														
Mean	37.13	36.91	41.81	38.59	39.83	39.68	36.60	37.00	37.72	38.71	28.33	38.38	0.054	
SD	6.83	7.91	2.613	6.48	4.589	7.89	5.50	7.19	5.69	6.74	2.08	6.15		
Negative professional identity subscale														
Mean	7.73	7.46	7.13	7.70	7.50	6.18	7.87	7.44	8.12	7.2	7.33	6.50	0.453	
SD	2.85	3.15	3.70	3.34	2.11	2.87	3.14	2.53	2.86	3.87	2.08	2.48		
Positive professional identity subscale														
Mean	11.69	11.73	13.50	11.87	12.58	12.64	12.133	11.83	11.78	12.82	9.33	12.44	0.065	
SD	2.50	2.69	1.59	2.63	1.16	2.87	1.85	1.69	2.18	2.21	0.58	2.34		
Roles and responsibilities subscale														
Mean	8.23	8.76	8.25	9.17	8.83	8.50	10.53	9.88	9.57	9.94	8.00	8.94	**0.001**	
SD	2.51	2.55	2.41	2.30	1.75	2.13	2.10	1.68	2.20	3.27	2.65	1.81		
Total RIPLS														
Mean	64.79	64.87	70.69	67.33	68.75	67.00	67.13	66.17	67.20	68.76	53.00	66.25	**0.042**	
SD	9.18	12.01	7.14	6.49	4.71	11.30	5.91	8.82	8.46	9.67	3.61	5.18		

*P*-value < 0.05 was considered statistically significant

Bold indicates statistically significant comparison

**Table 5:** Descriptive statistics of Revised Readiness for Interprofessional Learning Scale (RIPLS) across academic year

	Academic Year							
	First year	Second year	Third year	Fourth year	Fifth year	Sixth year	Seventh year	*P*-value*
Overall, % (n)	9.12 (106)	26.68 (310)	21.86 (254)	2.67 (31)	14.29 (166)	1.72 (20)	0.51 (6)	-
Student distribution %(n)	7.87 (44)	38.81 (189)	33.63 (188)	3.22 (18)	19.14 (107)	1.78 (10)	0.54 (3)	-
Teamwork and collaboration subscale								
Mean	38.82	38.31	36.89	40.83	36.35	40.00	37.00	**0.024**
SD	4.82	5.60	7.05	4.89	8.86	3.86	1.00	
Negative professional identity subscale								
Mean	6.77	7.56	7.76	6.83	7.85	7.20	9.00	0.347
SD	2.38	2.97	2.88	3.31	3.26	2.82	5.29	
Positive professional identity subscale								
Mean	12.11	12.06	11.58	12.44	11.91	12.60	11.33	0.394
SD	2.13	2.20	2.64	2.23	2.67	1.84	2.52	
Roles and responsibilities subscale								
Mean	8.93	9.32	8.43	8.61	8.45	8.50	9.67	**0.016**
SD	1.89	2.34	2.61	2.45	2.59	1.65	1.53	
Total RIPLS								
Mean	66.64	67.24	64.66	68.72	64.56	68.30	67.00	0.060
SD	5.75	8.36	9.96	5.22	11.42	4.30	3.61	

*P*-value < 0.05 was considered statistically significant

Bold indicates statistically significant comparison

** Calculated using the parametric One Way ANOVA Test

Only one RIPLS subscale was significantly different among HP programs (roles and responsibilities, *p* = 0.001) (Table 4). When RIPLS subscales were compared across academic years, teamwork and collaboration (*p* = 0.024), and roles and responsibilities (*p* = 0.016) subscales were shown to be significantly different. Second-year students had the highest score in roles and responsibilities subscale (*p* = 0.008) (Table 5).

Having previous experience with IPE was negatively correlated with total score of IEPS (*r* = −0.08, *p* = 0.033), and perceived need for cooperation subscale of the IEPS (*r* = −0.107, *p* = 0.007). On the other hand, having previous experience with IPE was negatively correlated with total score of RIPLS (*r* = −0.097, *p* = 0.022), teamwork and collaboration (*r* = −0.141, *p* = 0.001), and positive professional identity (*r* = −0.093, *p* = 0.027) subscales of RIPLS (Table 6).

**Table 6:** Correlation between scales and previous experience with IPE

	Point Biserial correlation	
Tools of each questionnaire	Correlation coefficient (rpb)	*p*-value^*^
IEPS		
Total score	−0.085	**0.033**
Perception of actual cooperation subscale	−0.073	0.065
Perceived need for cooperation subscale	−0.107	**0.007**
Competency and autonomy subscale	−0.058	0.147
RIPLS		
Total score	−0.097	**0.022**
Teamwork and collaboration subscale	−0.141	**0.001**
Negative professional identity subscale	0.080	0.058
Positive professional identity subscale	−0.093	**0.027**
Roles and responsibilities subscale	0.015	0.718

Bold indicates statistically significant comparison

Looking at the subgroup of participates that completed both questionnaires, participants that did not have previous IPE experience scored significantly higher on the perceived need for cooperation subscale of the IEPS (*p* = 0.020). the group that did not have previous IPE experiences also scored significantly higher on the teamwork and collaboration subscale of the RIPLS (*p* = 0.004) (Table 7).

**Table 7:** Comparison between scales and previous IPE experience in participants that completed both questionnaires

Questionnaire	IPE Experience (*N* = 166) (Mean ± SD)	No IPE Experience (*N* = 393) (Mean ± SD)	*p*-value*
IEPS (*N* = 559)			
Total score	50.8(13.7)	53.1(10.9)	0.062
Perception of actual cooperation subscale	20.7(6.4)	21.7(5.4)	0.074
Perceived need for cooperation subscale	8.2(2.5)	8.7(2.3)	**0.020**
Competency and autonomy subscale	21.9(6.2)	22.7(5.4)	0.170
RIPLS (*N* = 559)			
Total score	64.5(12.0)	66.5(8.0)	0.052
Teamwork and collaboration subscale	36.1(8.3)	38.2(6.0)	**0.004**
Negative professional identity subscale	7.9(3.3)	7.4(2.8)	0.076

*P*-value < 0.05 was considered statistically significant and highlighted in bold

Bold indicates statistically significant comparison

* calculated using independent samples t-tests

## Discussion

To our knowledge, this is the first study situated in the Middle East/Arabian Gulf region examining IPE attitudes and perception across a variety of HP students (MD, PT, OT, RS, MLS, ST, HI, NR, DS, Ph, and NI programs). A total of 1162 students completed the RIPLS and/or IEPS tools across 12 different health professions and 7 years of study. The results indicate that the students from all programs have positive attitudes toward IPE. Cross-sectional analyses revealed that total IEPS varied by year but not by program of study, while total RIPLS varied by program of study and not by year.

Total IEPS and RIPLS scores showed distinct patterns of variability. Specifically, the health professional program did not have an impact on student perception, whereas the year of study (i.e., Y1, Y2, etc.) showed variability, with students in advanced years scoring significantly higher than students in their early years as indicated by total IEPS scores (Table 1). These findings align with the way in which interprofessional experiences are facilitated in Kuwait through clinical exposure (e.g. participation in clinical rotations, rounds, and joint therapeutic sessions) and are only introduced in the third or fourth years across programs. Therefore, students in their early years regardless of their program of study will likely have similar attitudes towards the perceived importance of IPE due to the similar lack of interaction with other professions. In addition, all IEPS subscales measuring student perceptions were influenced by academic years (Table 3). The evidence of the timeliness of IPE exposure in HP programs has been mixed, with evidence for the importance of early exposure [17], evidence supporting later exposure [10], and evidence for no difference in time of exposure [8]. One such study conducted in Saudi Arabia (an approximate geographical region) reported that junior health sciences students from at least seven programs scored significantly higher on one IEPS subscale: Competency and Autonomy [3]. This finding has been attributed to having exposure to hospital rotations early on in their programs, providing an earlier opportunity for interaction with the different health professions. In contrast, our findings suggest that as students in Kuwait progress through their respective programs and gain more clinical experience, their perceptions of interdisciplinary collaboration evolve as well as their perceived mastery, likely due to increased exposure to clinical and hospital settings where teamwork and leadership are crucial.

In contrast to IEPS, total RIPLS scores varied across programs, indicating that the perception of readiness for interprofessional practice differed and was inherent to the program themselves and their curricula, rather than time spent and year of study in that program. For example, students in the ST program had the highest total RIPLS scores, whereas NM had the lowest. This may be attributed to the inherently collaborative nature of ST, which emphasizes interdisciplinary teamwork in clinical practice [16], in contrast to the more specialized and independent focus often associated with NM [6]. Similar to the findings for IEPS above, the literature on RIPLS and program-specific differences is mixed: some studies report significant variations across programs [1], while others find no such differences [14, 19]. Where differences are observed, they are often attributed to factors such as the collaborative structure of the curriculum, the extent of interprofessional exposure, and the cultural emphasis placed on teamwork within each profession, factors likely influencing the trends observed in this study.

Significant differences between HP majors/programs were observed in the RIPLS subscale scores related to roles and responsibilities specifically *Teamwork and Collaboration* along with *Roles and Responsibilities*. Notably, nursing students scored the highest in this domain, perhaps highlighting the emphasis placed on teamwork and interprofessional collaboration within their educational framework. The nursing program is the only health profession program with a geographic separation given they are offered by a different institution from the other HP programs, which could be the reason behind the students’ eagerness to learn more about other professions and interact with them. A similar trend was observed by Keshtkarana and colleagues where students of health professions scored significantly different on Teamwork and Collaboration (NR scoring the highest vs. MD scoring the lowest) and Roles and Responsibilities (MD scoring the highest vs. science in surgical technology scoring the lowest) in terms of RIPLS subscales [18]. Further, Alharbi and colleagues found a significant difference among health profession students in the *Negative Professional Identity* RIPLS subscale, with medicine scoring the highest in comparison to applied medical sciences scoring the lowest [2]. Similarly, a 10-year cross-sectional analysis of IPE evaluations in Canada demonstrated significant program-level differences in RIPLS subscales, particularly in Teamwork and Collaboration and Roles and Responsibilities, with nursing, physiotherapy, and physician assistant students consistently scoring higher than their peers in medicine [26]. These findings echo the trends observed in the present study and reinforce the idea that program structure, emphasis on interprofessional learning, and institutional context can shape students’ readiness for IPE. This could be an indicator that students in health professions programs in the Middle East exhibit readiness toward IPE in distinct ways.

A notable finding in our study was that students with no prior IPE experience scored higher on their perception and readiness in total RIPLS and IEPS than those with prior exposure. Although counterintuitive, this finding may suggest that initial IPE experiences may not be impactful or well-structured enough to positively influence attitudes toward interprofessional learning. This could also be caused by the novelty effect, which might inflate inexperienced students’ perception toward IPE compared to students with prior experience with IPE [24]. Similar trends were also observed with participants that completed both questionnaires, which could also indicate optimism and excitement of non-exposed students toward future IPE experiences. These findings underscore the importance of high-quality, formative IPE experiences in shaping student perceptions and attitudes [11]. The negative correlation observed between having previous IPE experience and subscales of IEPS (i.e. total score and Perceived Need for Cooperation subscale) and RIPLS (i.e. total score, Teamwork and Collaboration subscale, and Positive Professional Identity subscale) suggests that these aspects of interprofessional learning may require more structured or impactful IPE interventions to achieve meaningful change [4, 13].

Because the participants had the option to complete one or both questionnaires in this study, there was a discrepancy in the response rate of the participants. IEPS had better response rate (i.e. 54.7%) compared to RIPLS which may have been due to an order effect where the IEPS was the first questionnaire presented to the participants. This could also introduce differential non-response bias [21, 23]. To account for this type of bias, we analyzed the responses for the questionnaires for the participants that only completed said questionnaire (Tables 2, 3, 4 and 5). We also conducted further analysis on the participants that completed both questionnaires in terms of previous IPE experience status (Table 7).

### Strengths, limitations, and future directions

This study provides valuable insights into student perceptions of IPE in Kuwait, highlighting key factors that shape attitudes and readiness for collaborative practice. A major strength of the study lies in its large sample size (*n* = 835) and its inclusion of students from 12 distinct health professions and 7 different academic levels, offering a broad and diverse perspective from an understudied population of learners. Additionally, the use of validated tools such as the RIPLS and IEPS ensures reliability and comparability to the existing literature in other well studied contexts. The study also contributes to the current literature through its detailed analyses of trends across academic years and programs, contributing to a nuanced understanding of how clinical exposure and curriculum structure influence IPE perceptions.

However, several limitations should be noted. The cross-sectional design of the study captures a snapshot of student attitudes at a single point in time, limiting the ability to infer causality or track changes over the course of their programs. Also, the sample did not balance for the 12 majors nor years by program equally. The sample demographics also revealed a skewed gender distribution, with three-quarters of participants identifying as female, which may not fully represent the perspectives of male students or allow for a comprehensive gender-based analysis of interprofessional views. Future research exploring how interprofessional attitudes vary across genders could provide a more balanced perspective on the impact of gender on IPE. Finally, not all the students completed both tools (IEPS *n* = 636; 54.73%/RIPLS *n* = 559; 48.11%) which may have introduced bias and thus not have captured the broad insight of each student.

Future research should aim to address these limitations by incorporating longitudinal designs, ensuring balanced demographic representation, and exploring the role of curriculum-specific factors in shaping interprofessional attitudes. Further exploration of this concept using a qualitative method would provide a clearer picture of their perception and readiness toward IPE. The results will help policy makers plan a Kuwait-tailored IPE through guiding curriculum development in higher education for health-related institutions. This would help prepare competent and collaborative healthcare professionals. Constant assessment of the effectiveness of the IPE implementation would also be needed to ensure students engagement and satisfaction.

## Conclusion

To the best of our knowledge, this is the first study to assess the perception and readiness of HP students toward IPE in the region that included this divert of programs and through the years of study in each program. This study highlights the generally positive attitudes of students in Kuwait toward IPE, with notable variations influenced by academic level and program of study. Increased student perceptions of IPE among upper-year students emphasizes the role of clinical exposure in enhancing perceptions of interdisciplinary collaboration. Program-based variations indicate differences in curricula and exposure to IPE from one program to another. Creating a unique entity that would create and coordinate IPE events for all HP programs could be the solution for these variations.

The findings support each program in modifying their IPE curriculum to shape readiness for interprofessional practice. These findings underscore the need for structured IPE experiences tailored to student academic stages and professional contexts. Addressing these gaps through improved programming and targeted IPE interventions can better prepare students for collaborative healthcare environments. Educators would benefit from tailored IPE activities within their curriculum to encourage better engagement. Future research should explore longitudinal approaches and balanced demographics to refine IPE programs and foster more inclusive, impactful learning experiences.

Bold indicates statistically significant comparison.

## Data Availability

The data that support the findings of this study are available from the corresponding author upon request. Data are located in controlled access data storage at Kuwait University.

## Abbreviations

IPE: Interprofessional education
HP: Healthcare professions
IEPS: The Interdisciplinary Education Perception Scale
RIPLS: Readiness for Interprofessional Learning Scale
HSC: Health Science Center
MD: Medicine
PT: Physical Therapy
OT: Occupational Therapy
RS: Radiological Sciences
MLS: Medical Laboratory Sciences
ST: Speech Therapy
HI: Health Informatics
NR: Nursing
DS: Dentistry
Ph: Pharmacy
NI: Nutrition
KU: Kuwait University
